# Role of P2X_4_ Receptor in Mouse Voiding Function

**DOI:** 10.1038/s41598-018-20216-4

**Published:** 2018-01-30

**Authors:** Weiqun Yu, Warren G. Hill, Simon C. Robson, Mark L. Zeidel

**Affiliations:** Department of Medicine, Beth Israel Deaconess Medical Center and Harvard Medical School, Boston, Massachuesetts USA

## Abstract

Purinergic signalling plays an important role in the regulation of bladder smooth muscle (BSM) contractility, and P2X_4_ receptor is expressed in the bladder wall, where it may act by forming heteromeric receptors with P2X_1_, the major purinergic force-generating muscle receptor. To test this hypothesis, we examined mouse BSM contractile properties in the absence and presence of selective P2X_1_ (NF449 & NF279) and P2X_4_ antagonists (5-BDBD). These drugs inhibited BSM purinergic contraction only partially, suggesting the possibility of a heteromeric receptor. However, carefully controlled co-immunoprecipitation experiments indicated that P2X_1_ and P2X_4_ do not form physically linked heteromers. Furthermore, immunofluorescence staining showed that P2X_4_ is not present in mouse BSM *per se*, but in an unknown cellular structure among BSM bundles. To investigate whether deletion of P2X_4_ could impact voiding function *in vivo*, P2X_4_ null mice were characterized. P2X_4_ null mice had normal bladder weight and morphology, normal voiding spot size and number by voiding spot assay, normal voiding interval, pressure and compliance by cystometrogram, and normal BSM contractility by myography. In conclusion, these data strongly suggest that P2X_4_ is not present in mouse BSM cells, does not affect smooth muscle contractility and that mice null for P2X_4_ exhibit normal voiding function.

## Introduction

The prevalence of lower urinary tract symptoms (LUTS) is extremely high, affecting ~50% of the population aged more than 40 years old^1,2^, and the major symptoms of LUTS are manifested by bladder contraction (voiding) and relaxation (storage) disorders. Mechanistic understanding of LUTS is still unclear, however, purinergic signalling is a major pathway in modulating bladder physiology/pathophysiology. In normal human bladders, atropine blocks ~95% of nerve-mediated bladder smooth muscle (BSM) contraction (induced by electrical field stimulation-EFS), leading many to conclude that purinergic contractility is insignificant. However, a deeper look at the literature reveals controversy and unresolved questions: the neuromuscular purinergic component in female bladders and in the bladder trigone accounts for about 50% and 40% of nerve-mediated contraction force respectively^3,4^. Furthermore, externally applied α,β-meATP (as opposed to EFS-stimulated neuronal release) elicited significant human BSM contraction, and purinegic receptors like P2X_1_ is highly expressed in human bladder^5,6^. In human bladder with pathological conditions, contraction in response to neuromuscular purinergic signalling can account for up to 65% of total force in patients with overactive bladder, partial bladder outlet obstruction, and diabetic bladder dysfunction (DBD)^7–12^, and elevated ATP release/altered P2X receptor expression have been reported in these patients. The importance of purinergic signalling in regulating bladder function is further supported by recent findings from two human patients with ectonucleotidase ENTPD1 mutation, who display bladder hypomotility and incontinence, in addition to neurological conditions^13^.

Current evidence on purinergic component or atropine-resistant contractile force in BSM contraction favours a dominant role for P2X_1_. First, P2X_1_ receptor is the major purinergic receptor expressed in BSM. Second, BSM from P2X_1_ deficient mice lose the majority of their purinergic force, which is perhaps unsurprising since P2X_1_ is believed to initiate the rapid contraction phase in BSM^14^. However, there is additional important evidence that purinergic receptors other than P2X_1_ play an important role. First, RT-PCR or immunoblotting studies reveal that multiple P2X receptors are expressed in bladder^15–18^. Second, in guinea pig bladder, significant neurogenic contractions can be elicited even in the presence of cholinergic and P2X_1_ receptor antagonists^19^. Third, in diabetic rat models like streptozotocin (STZ) treatment or Zucker obese rats, bladders exhibited significant non-cholinergic and non-α,β-meATP sensitive contractions accounting for 20% of total nerve mediated contractile force^20,21^. These results suggest that there are additional P2 receptors active in BSM and indeed abundant expression of P2X_4_ has been observed by immunostaining in BSM as well as in other smooth muscle^22^. Since P2X receptors are trimeric and in many tissues, heteromeric (with mixed P2 subtypes), this raises the question of whether P2X_4_ forms either functional homomers or possibly functional heterotrimers with P2X_1_ and thereby participates in BSM contraction^23^.

To determine the potential role of P2X_4_ in BSM function, we have examined BSM contraction in the absence and presence of P2X_4_ antagonists. We have also used co-immunoprecipitation techniques to examine whether P2X_4_ forms heteromers with P2X_1_. Finally we have determined the role of P2X_4_ in overall voiding function by studying a P2X_4_ knockout mouse.

## Results

### Pharmacological characterization does not support a P2X_1_ homomer in BSM

**Figure 1:**
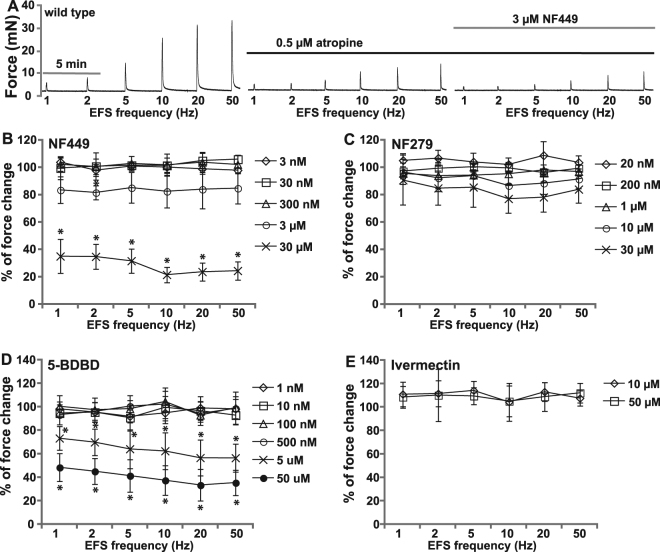
Pharmacological properties of BSM contractility. BSM strips were stimulated by EFS at indicated frequencies in increasing order ((**A**)-left). Raw data showing effect of sequential addition of atropine and NF449 (pre-treatment 15 min) on EFS force ((**A**)-right panels). Selective P2X_1_ antagonists NF449 ((**B**), n = 7), NF279 ((**C**), n = 4), or selective P2X_4_ antagonist 5-BDBD ((**D**), n = 7), or P2X_4_ potentiator, Ivermectin ((**E**), n = 8) were then added at indicated concentrations (with accumulative addition) for 15 min to measure the atropine-resistant force change in BSM contraction in response to EFS. Force changes were normalized to control and shown as percentages. These data indicate that P2X_1_ and P2X_4_ antagonists only partially inhibit BSM atropine resistant force at relatively high concentrations. Ivermectin, a P2X_4_ potentiator, does not increase BSM atropine resistant force significantly. BSM atropine resistant force before drug treatment was used as control (100%), and drug effect (inhibition or potentiation) was normalized to control. Data were analysed using one-way ANOVA and then Bonferroni’s multiple comparison post-hoc tests. * indicates *P* < 0.05 when compared to control.

During electric field stimulation (EFS), BSM contraction is stimulated through cholinergic and non-cholinergic (or purinergic) neurotransmitter release. In the presence of atropine, cholinergic stimulation is eliminated, permitting assay of atropine resistant or purinergic contraction. P2X_1_ is sensitive to α,β-meATP, but many other P2X receptors, including some P2X heteromers are also sensitive to α,β-meATP^24–26^. Potent and more selective P2X_1_ antagonists such as NF449 (IC50 = 0.28 nM) and NF279 (IC50 = 19 nM) are now well documented^24^. We therefore examined the ability of these antagonists to alter the contraction force developed by BSM strips, in response to EFS in the presence of sufficient atropine to block the cholinergic response. As shown in Fig. 1, these P2X_1_ antagonists were only marginally effective in attenuating EFS-induced, atropine-resistant contractions (Fig. 1B,C). NF449 did inhibit BSM contraction significantly at a concentration of 30 µM, a level 10^5^ times higher than the IC_50_, at which selectivity is lost and the antagonist inhibits most P2X subunits (Fig. 1B). NF279 did not show any significant inhibition on BSM contraction even when the concentration reached 30 µM (Fig. 1C). P2X_4_ is a unique P2X receptor which does not show sensitivity to most P2X agonists and antagonists^24^. 5-BDBD (IC50 = 0.5 µM) might be the most potent P2X_4_ antagonist available. We showed that it was able to inhibit EFS induced atropine-resistant BSM contractions at concentrations between 0.5–50 µM (Fig. 1D), indicating that P2X_4_ might be involved in BSM contraction. By contrast, ivermectin (EC_50_ = 0.2 µM), a large macrocylic lactone that is known to potentiate P2X_4_ channel current did not increase EFS-induced atropine-resistant BSM contraction at concentrations as high as 50 µM (Fig. 1E). In summary, there were weak effects using the specific antagonists alone, suggesting that BSM might contain a pharmacologically unique heteromeric P2X_1_ trimer, and that P2X_4_ could participate as a subunit of the trimer.

### P2X_1_ and P2X_4_ heteromers were not detectable by immunoprecipitation

**Figure 2:**
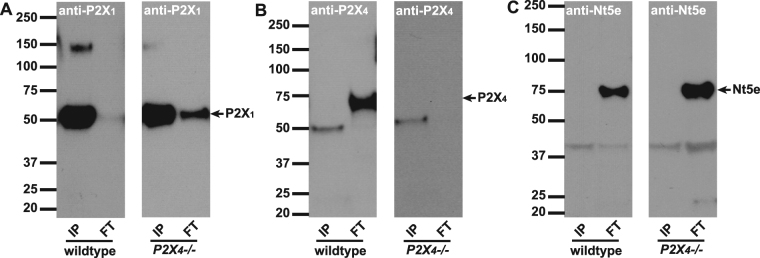
Immunoprecipitation with anti-P2X_1_ antibody. Antibodies to P2X_1_ were immobilized onto resin beads and then incubated with mouse bladder lysates to IP the antigen and co-IP interacting proteins. Proteins that were bound (IP: 2.5 µg protein/lane) or did not bind (FT: 25 µg protein/lane) to the beads were resolved by SDS-PAGE, and Western blots were probed with A) P2X_1,_ B) P2X_4_ or C) Nt5e antibodies. (**A**) Left and right panels show P2X_1_ immunoblots on IP and FT lysates from wild type and *P2X*_4_*−/−* mice. Monomeric P2X_1_ can be seen highly concentrated in the pulldown fraction at 50 kDa. Little P2X_1_ appears in FT. (**B**) P2X_4_ antibody detects P2X_4_ as a band at 70 kDa in wild type, but is completely absent in *P2X*_4_*−/−* mice. The antibody shows minor cross-reactivity to possibly P2X_1_. Note, there is no evidence of the 70 kDa band in the IP lane. (**C**) An antibody to 5′-nucleotidase (Nt5e) demonstrates that pulldown with anti-P2X_1_ is ‘clean’ with no non-specific protein binding evident in the IP lanes.

To test the hypothesis suggested by the pharmacology, we attempted to co-immunoprecipitate both proteins. The specificity of the anti-P2X_1_ antibody we used, has been validated previously on bladder tissues from a knockout mouse^27,28^, and our results demonstrate that a specific protein band at around 50 kD becomes highly enriched when bladder protein lysate is immunoprecipitated with it (Fig. 2A). This was true for bladders from wild type as well as P2X4 null mice. In the right panel of Fig. 2A we see evidence of a small amount of unbound P2X1 in the flow-through (FT) fraction. No protein is immunoprecipitated when the antibody is omitted from the pull-down (data not shown), indicating that detected protein is specifically bound to the antibody.

We performed immunoprecipitation with the anti-P2X_1_ antibody to examine whether P2X_4_ protein could be co-immunoprecipitated along with P2X_1_ (Fig. 2B). The anti-P2X_4_ antibody has also been validated previously for its specificity, using knockout mice^29^. P2X_4_ protein (~70 kD) was only detected in the non-bound FT samples of wild type mice (Fig. 2B, left panel). There was no ~70 kD band in the IP lane. Strikingly, the positive identification of P2X_4_ in the flow through was confirmed by its absence in P2X_4_ knockout samples (Fig. 2B, right panel). There was in addition a band at detected ~50 kD by P2X_4_ antibody. Since it appears in the co-IP’d lanes of both wild type and *P2X*_4_*−/−* tissue and is the same molecular size as P2X_1_, there is a very strong likelihood that the P2X_4_ antibody shows minor cross-reactivity to P2X_1_.

As a further control for the specificity of the co-IP we blotted P2X_1_ pulldown samples with anti-Nt5e antibody, an enzyme that converts AMP to adenosine and which we have identified in BSM previously^30^ (Fig. 2C). The results showed that Nt5e protein was only present in non-bound FT samples but was completely undetectable in co-IP’d protein samples (Fig. 2C). Thus the data indicates that despite strong P2X_1_ pulldown, there is no P2X_4_ and Nt5e associated with it.

**Figure 3:**
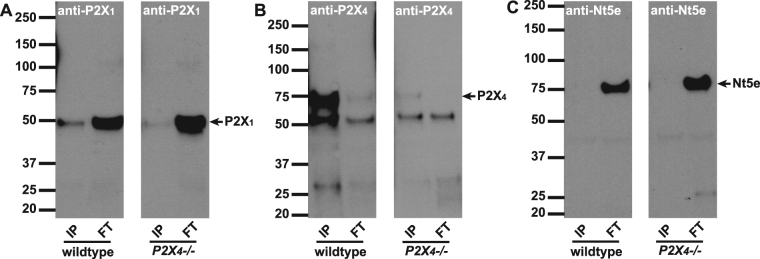
Immunoprecipitation with anti-P2X_4_ antibody. Antibodies to P2X_4_ were immobilized onto resin beads and then incubated with mouse bladder lysates to IP the antigen and co-IP interacting proteins. Proteins that were bound (IP: 2.5 µg protein/lane) or did not bind (FT: 25 µg protein/lane) to the beads, were resolved by SDS-PAGE, and Western blots were probed with **A**) P2X_1,_ B) P2X_4_ or **C**) Nt5e antibodies. (**A**) Left and right panels show P2X_1_ immunoblots on IP and FT lysates from wild type and *P2X*_4_*−/−* mice. P2X_1_ is highly concentrated in the FT fractions. Minor potential P2X_1_ staining appears in the IP lane, however this is due to P2X_4_ antibody cross-reacting and pulling down some P2X_1_. (**B**) P2X_4_ antibody detects P2X_4_ as a band at 70 kDa in wild type IP lane, but is absent in *P2X*_4_*−/−* mice. The antibody shows minor cross-reactivity to P2X_1_ (50 kDa band). (**C**) An antibody to 5′-nucleotidase (Nt5e) demonstrates that pulldown with anti-P2X_4_ is ‘clean’ with no non-specific protein binding evident in the IP lanes.

We then performed the converse experiment with co-immunoprecipitation by anti-P2X_4_ antibody (Fig. 3). As judged by Fig. 3B, P2X_4_ protein (70 kD) was highly accumulated in IP fractions from wild type bladder but which disappeared in P2X_4_ knockout samples. P2X_1_ protein was highly concentrated in non-bound FT samples, but was detectable, albeit lightly in co-IP fractions (Fig. 3A). Given that the P2X_4_ antibody is likely to have some cross-reactivity to P2X_1_ the presence of weak bands in the IP fractions of Fig. 3A,B are to be expected. This muddies the interpretation somewhat, due to one imperfect antibody, however, if one accepts the high likelihood that anti-P2X_4_ cross-reacts minimally with P2X_1_, taken together data from these reverse IP experiments supports the conclusion that P2X_1_ and P2X_4_ do not form functional trimers. The overall optimization of conditions to ensure ‘clean’ pulldowns by bead-linked antibodies, is confirmed by Nt5e immunoblot in the P2X_4_ pulldown experiment (Fig. 3C).

### P2X_4_ is expressed in bladder, but not in the BSM

**Figure 4:**
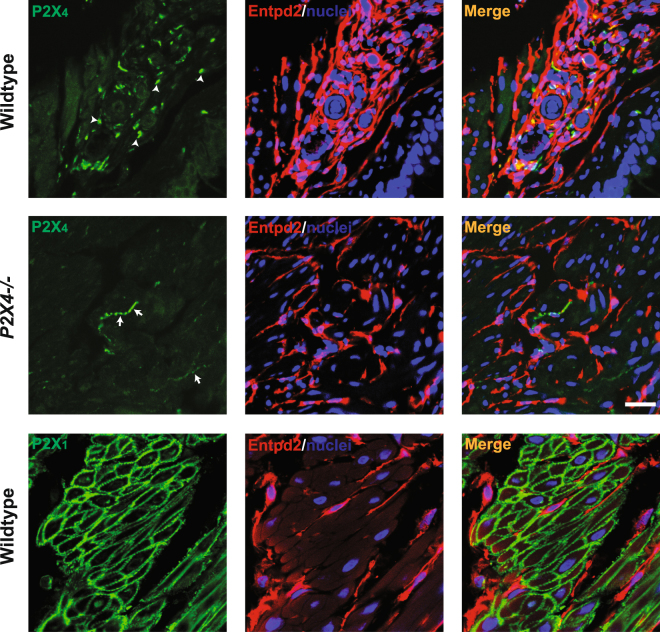
P2X_4_ is expressed in bladder wall but not in BSM. Cryosections of mouse (wild type and *P2X*_4_*−/−*) bladders were labelled with antibodies to P2X_4_ (green), P2X_1_ (green), ENTPD2 (red), and Topro-3 to label nuclei (blue). Entpd2 (middle panels) labels interstitial cells among muscle bundles. Colour merged panels are shown on the right and merged signals are seen as yellow. White arrowheads (top left) indicate P2X_4_ labelling of unknown cellular structures. White arrows (middle left) indicate non-specific labelling by anti-P2X_4_ antibody in *P2X*_4_*−/−* mice. P2X_1_ receptors are abundantly expressed in BSM and that signal is clearly differentiated from interstitial cell staining (red). White scale bars = 10 µm.

P2X_4_ was reported to be abundantly expressed in smooth muscle, including in BSM^22,31^. It was also reported that P2X_1_ and P2X_4_ can form functional heterotrimers in both native and artificial systems^25,31–34^. Our co-IP data did not support a P2X_1_/P2X_4_ heteromer in the bladder. Further support for this conclusion was provided by immunofluorescent staining and confocal imaging, which indicated that P2X_4_ is expressed in the bladder wall but is not in smooth muscle itself (Fig. 4). The left panels (top) show discrete punctate P2X_4_ staining in wild type bladder (white arrowheads) and these appear to be true P2X_4_ positive cells because that unique staining pattern is absent in *P2X*_4_*−/−* bladders (left middle panel). There is however non-specific labelling of thin fibrous structures in the P2X4 knockout (white arrows).

Entpd2 (in red) is an ectonucleotidase that we have shown is specifically expressed in interstitial cells that tend to wrap around smooth muscle bundles in bladder^30^. From the merged panel it appears that P2X_4_ positive cells are detected between muscle bundles and some are possibly associated with vascular elements. To our surprise, we were unable to detect a significant P2X_4_ signal in BSM. However, BSM was strongly labelled by P2X_1_ antibody (Fig. 4 bottom green), which is consistent with our Co-IP data and previous reports. In an attempt to reconcile our data with previous reports of strong P2X_4_ staining^22,35^ we used the same commercially available anti-P2X_4_ antibody (Enzo Life Sciences: catalogue #: Alx-215-033-R100). Non-specific protein bands (western blot) and a strong immunofluorescent signal were detected in BSM of both wildtype and *P2X*_4_*−/−* mice using this antibody (Supplemental Figure 1), thus indicating that these earlier reports may have been misled as a result of poor antibodies.

To further define the non-specific immunostaining of thin fibers in the P2X_4_ knockout we co-labeled cryosections with anti-PGP9.5, anti-β1 integrin, and anti- PDGFRα antibodies. Our results indicate that the fibrous structures do not co-localize with either β1 integrin signalling, which stains BSM strongly, or interstitial cell marker PDGFRα. They do however, co-label with nerve marker PGP.9.5, indicating those structures are very likely to be sensory nerve fibers, since P2X_2/3_ has well documented expression there^36^ (Supplemental Figure 2). In summary these data indicate that P2X_4_ is expressed in bladder wall but not in BSM cells.

### Mice null for P2X_4_ do not exhibit abnormal urinary function or abnormal BSM contractility

**Figure 5:**
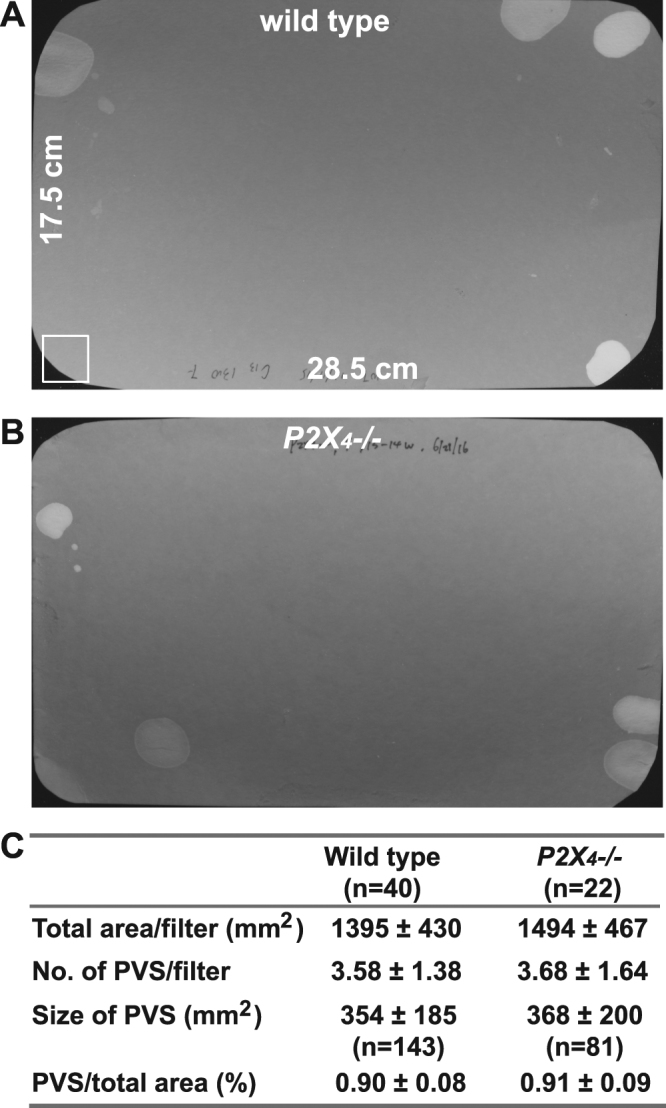
*P2X*_4_*−/−* mice exhibit normal voiding behaviour by void spot assay. Representative filters showing urine spots under UV light from a wild type mouse (**A**) and a *P2X*_4_*−/−* mouse (**B**). Quantitative data are shown in (**C**) and indicates *P2X*_4_*−/−* mice have normal voiding volume, spot number, and spot size. The square at bottom left of panel (**A**) (surface area of 400 mm^2^) serves as a size standard.

*P2X*_4_*−/−* mice were further used to evaluate whether P2X_4_ plays a role in regulating voiding function. Both male and female *P2X*_4_*−/−* mice have similar body weights to wild type mice. Their bladders are also visually normal with the weight in the normal range (Table 1). Voiding spot assay indicates that these mice have normal voiding volume, voiding spot numbers, and voiding spot size per void (Fig. 5).

**Table 1:** *P2X*_4_*−/−* mice have normal body and bladder weights.

	Male		Female	
	Wild type (n = 7)	*P2X*_4_*−/−* (n = 7)	Wild type (n = 8)	*P2X*_4_*−/−* (n = 6)
Body weight (g)	26.83 ± 2.67	27.39 ± 2.51	20.75 ± 2.10	21.83 ± 1.88
Bladder weight (mg)	26.28 ± 2.41	27.11 ± 2.17	20.78 ± 1.74	21.20 ± 2.45
Bladder/body ratio (mg/g)	0.98 ± 0.08	0.99 ± 0.06	1.01 ± 0.04	0.97 ± 0.07

**Figure 6:**
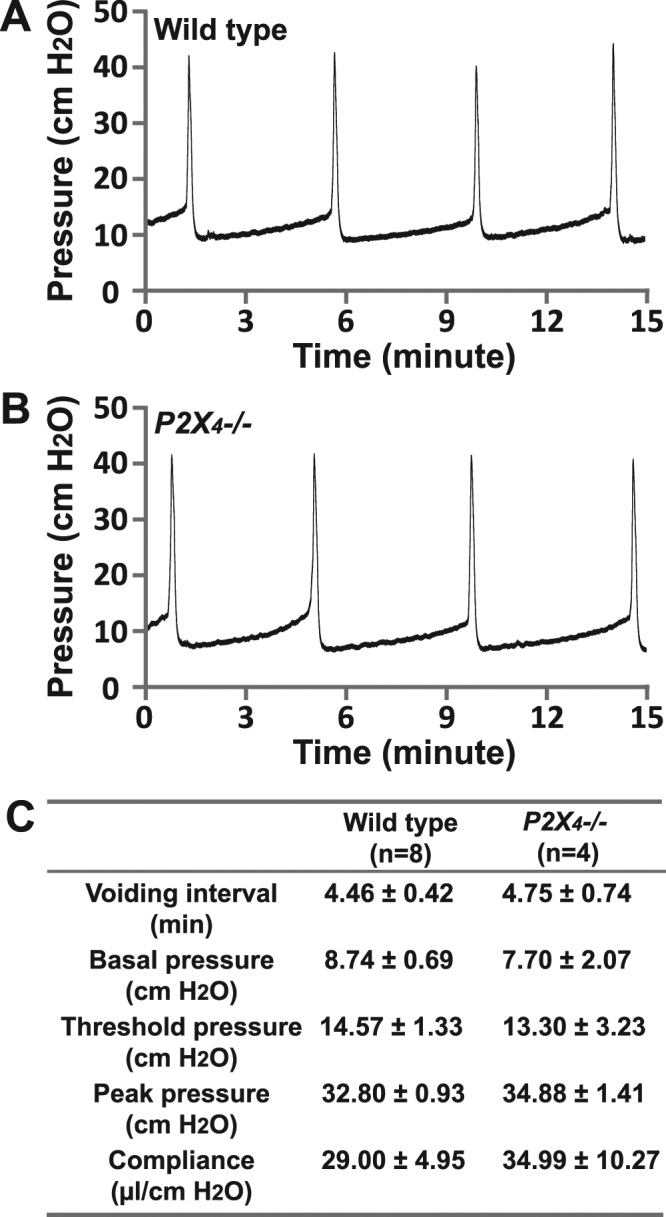
Cystometrograms showing intrabladder pressure throughout multiple filling/emptying cycles indicate that *P2X*_4_*−/−* mice have normal voiding interval, pressure, and bladder compliance; (**A**) CMG trace for a wild type mouse; (**B**) CMG trace for a *P2X*_4_*−/−* mouse; (**C**) Quantitation of cystometric parameters showing no significant differences between wild type and *P2X*_4_*−/−* mice.

**Figure 7:**
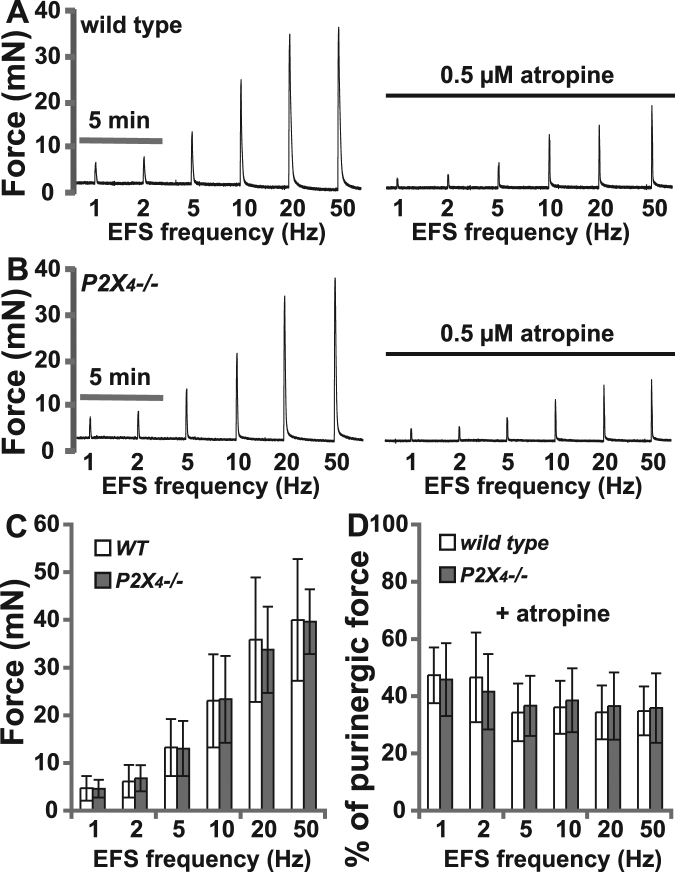
Myography indicates that BSM from *P2X*_4_*−/−* mice have normal contractility in response to electrical field stimulated neurotransmitter release. (**A**) Frequency train data on wild type bladder strips before and after atropine treatment; (**B**) Frequency train data on *P2X*_4_*−/−* bladder strips before and after atropine treatment; (**C**) and (**D**) Quantitation of force from wild type (n = 12) and *P2X*_4_*−/−* (n = 12) muscle strips. Data shown is mean ± SD.

We next performed cystometrograms on *P2X*_4_*−/−* mice (Fig. 6), and none of the analysed cystometric parameters showed any significant differences compared to wild type mice. These included voiding interval, basal pressure, micturition threshold pressure, peak pressure, and bladder compliance (Fig. 6C), thus confirming that *P2X*_4_*−/−* mice have undetectable changes of voiding function. Likewise, myography studies indicate that urothelium denuded BSM strips from *P2X*_4_*−/−* mice have normal contractility in response to EFS, including both the muscarinic and atropine resistant force components (Fig. 7). In summary, these data suggest that P2X_4_ does not contribute to physiological voiding function in mice.

## Discussion

P2X_4_ is reported to be expressed in many types of cells including glial cells in nerve tissue, vascular endothelial cells, macrophages, T lymphocytes, smooth muscle cells, and some epithelial cells^31^. In human vascular endothelial cells, P2X_4_ channels mediate ATP-induced calcium influx in response to fluid shear stress^37–39^. This mechanism is crucial in regulating blood pressure and vascular remodelling, and mice null for P2X_4_ exhibit higher blood pressure with reduced nitric oxide secretion^40^. P2X_4_ is well recognized to be involved in both acute and chronic pain responses. Upon injury or inflammation, microglial P2X_4_ is up-regulated, which mediates increased secretion of brain-derived neurotrophic factor (BDNF) and phosphorylation of Src family kinases Lyn, and these pathways are critical for neuropathic pain sensation^41–46^. Consistently, reduced pain responses and altered hippocampal synaptic potentiation have been observed in P2X_4_ null mice^47,48^.

The presence of P2X_4_ receptors in the bladder wall was noted long ago, and a quantitative analysis of transcripts indicated that it was the second most abundant P2X receptor (after P2X_1_) in normal human bladder wall^15,16,18,22,49^. P2X_4_ has been identified in BSM and lamina propria by immunolocalization, however, its functional role has not been studied yet. Interestingly, it has been observed that P2X_4_ is significantly up-regulated in rabbit bladders upon ischemia and oxidative stress, and in the bladders of patients with symptomatic outlet obstruction, suggesting a potential role in the pathogenesis of bladder dysfunction^17,50^.

In this study, we have confirmed that atropine resistant BSM contraction is insensitive to selective and potent P2X_1_ antagonists NF279 and NF449, but seems partially sensitive to P2X_4_ antagonist 5-BDBD (Fig. 1). This unique characterization of BSM has been noticed by several other studies, and a recent review article has provided a comprehensive summary^23^. Briefly, BSM atropine resistant force is partially α,β-meATP sensitive, and P2X antagonists like reactive blue 2, PPADS, suramin, and P2X_1_ selective antagonists NF279, NF449, MRS2159 are not very effective or even have no effect in inhibiting this atropine resistant force^23^. Interestingly, P2X_4_ is a unique P2X member which is not inhibited by reactive blue 2, PPADS, or suramin^51^, thus it was recently proposed that P2X_1_ and P2X_4_ might form heteromers in BSM^23^. Our pharmacological results are consistent with previous reports, and in combination with earlier morphological data showing strong P2X4 staining of BSM, led us to hypothesize that P2X_4_ in the BSM might form a functional heteromer with the dominant P2X_1_.

Functional heteromeric P2X_1_ and P2X_4_ receptors have been suggested in both artificial *Xenopus* oocytes expression system and native mouse macrophages and T lymphocytes^25,32–34^. This heteromeric channel is sensitive to α,β-meATP and PPADS, but it is not very sensitive to suramin and can only be partially inhibited at high concentration. These pharmacological properties in other cellular systems resemble closely the BSM response to these drugs, supporting our hypothesis. To our surprise, the co-IP data clearly indicated that P2X_1_ and P2X_4_ do not form heteromers in mouse bladder at all (Figs 2 and 3), and furthermore, our IF and imaging data indicate that P2X_4_ is not present in BSM cells, but in a small unknown cellular structure that is dispersed within muscle bundles, and in particular associated with circular structures that might be vasculature (Fig. 4). We do not know the identity of this P2X_4_ positive structure, but it could relate to neuronal structures, which are thought to have interactions with ENTPD2 positive interstitial cells located among muscle bundles^52^. It is possible that P2X_1_ forms homomeric receptors in BSM as suggested by P2X_1_ null mice, in which the BSM loses the majority of its purinergic force^14^. However, the potential existence of other P2X receptors, the non-typical P2X_1_ pharmacological properties of BSM, and the atropine resistant and α,β-meATP insensitive force remains unexplained and requires further investigation.

Although P2X_4_ is not present in BSM, its presence in the bladder wall might indicate that it plays some other functional role in regulating voiding. To examine this, we carefully characterized the voiding phenotype of P2X_4_ null mice in multiple complementary ways, and our results indicate that bladders are not macroscopically different, their voiding function appears completely normal according to voiding spot assay and cystometrogram data, and the BSM exhibits normal overall as well as purinergic contractility (Fig. 7). These data collectively indicate that P2X_4_ might not be a functional receptor for bladder contractility. It remains possible that P2X_4_ could play a role in the pathology of bladder diseases such as in bladder pain sensation, which remains a poorly understood area.

## Materials and Methods

### Materials

Unless otherwise specified, all chemicals were obtained from Sigma (St. Louis, MO) and were of reagent grade or better. Agonists and antagonists for P2X receptors were all purchased from R&D systems (Minneapolis, MN). All data generated or analyzed during this study are included in this published article.

### Animals

Male and female C57BL/6 J mice (Jackson Laboratory, Bar Harbor, ME, USA) and *P2X*_4_*−/−* mice in C57BL/6 J background (kindly provided by Dr. Francois Rassendren, CNRS, France) (aged 12–16 weeks) were used in this study with the approval of the Beth Israel Deaconess Medical Centre Institutional Animal Care and Use Committee. Animals were used in adherence to NIH guidelines. All experiments and groups were performed under matching conditions for age and sex. Only male or female mice were used in some experiments (see below). If not specified, both male and female mice were used. Mice were euthanized by 100% CO_2_ inhalation from a gas cylinder into a plexiglass chamber.

### Myography

Bladders from male wild type and *P2X*_4_*−/−* mice were pinned on a small Sylgard block and bladder mucosa was dissected away carefully. BSM strips were then cut longitudinally (2–3 mm wide and 5–7 mm long) and mounted in an SI-MB4 tissue bath system (World Precision Instruments, FL, USA). Force sensors were connected to a TBM 4 M transbridge and the signal amplified by Powerlab and monitored through Chart software. Contraction force was monitored dynamically with a sampling rate of 2000/s. BSM strips were gently pre-stretched to get optimized force and equilibrated for at least 1 h before any experiments. All experiments were conducted at 37 **°**C in physiological saline solution (PSS in mM: Na, 136.9; K, 5.9; Ca, 2.5; Mg, 1.2; Cl, 133.6; HCO_3_, 15.5; H_2_PO_4_, 1.2; glucose, 11.5; pH 7.4), with continuous bubbling of 95% O2 and 5% CO2.

### Electrical field stimulation (EFS)

EFS was carried out by a Grass S48 field stimulator (Grass Technologies, RI, USA) using standard protocols previously described^53^.

### Co-Immunoprecipitation

Anti-P2X_1_ (Catalogue #: APR-001, Alomone Labs) and anti-P2X_4_ antibody (Catalogue #: APR-002, Alomone Labs) were pre-cleaned by Pierce Antibody Clean-up Kit (Thermo Fisher Scientific) for co-immunoprecipitation according to the manufacturer’s instructions. Antibodies were then immobilized onto amine-reactive resin beads to IP the antigen and co-IP the interacting proteins using the Pierce Co-Immunoprecipitation (Co-IP) Kit (Thermo Fisher Scientific) to isolate protein complexes from native mouse bladder lysate according to the manufacturer. The isolated proteins were resolved on 8–16% polyacrylamide gradient gels under reducing condition (+0.1 M DTT and 95 °C for 5 min before loading), and further blotted and probed with anti-P2X_1_, anti-P2X_4_ and anti-5′-nucleotidase (NT5E) (Catalogue #: MAB44881, R&D system) antibodies for protein detection. If P2X_1_ and P2X_4_ form heteromers, pull-down of P2X_1_ protein by P2X_1_ antibody will also pull-down P2X_4_, and vice versa.

### Western Blot

Excised whole bladders were put in 0.5 ml ice-cold radio immunoprecipitation assay buffer (RIPA; 50 mM Tris pH 8.0, 150 mM NaCl, 1% v/v NP-40, 0.5% w/v deoxycholic acid, 0.1% w/v SDS) containing Complete Mini Protease Inhibitor Cocktail tablets (Roche, Germany). Proteins were resolved by SDS-PAGE (Tris-HEPES 8–16% gel, catalogue #: NH11-816; NuSep, GA) in Tris-HEPES running buffer (12.1 g Tris, 23.8 g HEPES, 1.0 g SDS, and H_2_O to 1000 ml) at 120 constant voltage for 45–60 min, and then transferred to Immun-Blot PVDF membrane (BioRad Laboratories, Hercules, CA) in transfer buffer (Tris base 3.0 g, bicine 4.08 g, methanol 100 ml, and H_2_O to 1000 ml) at 350 mA for 90-120 min at 4 °C. The blots were blocked in 5% dehydrated milk in PBS overnight at 4 °C, and then were probed with specific antibodies in 1% dehydrated milk in PBS for 2 hours at room temperature, followed by the appropriate species-specific secondary antibodies conjugated to HRP for 1 hour at room temperature. Three time 15 min washes were performed after the first and the secondary antibodies incubation with TBS Tween 20 (0.05%). Bands were detected using ECL Plus Western Blotting reagents (GE Healthcare, Piscataway, NJ) and CL-X Posure film (Thermo Scientific, Rockford, Il). The film was developed, scanned and images were imported into Adobe Illustrator CS3 (San Jose, CA).

### Immunofluorescence (IF) staining and confocal microscopy

Both wild type and *P2X*_4_*−/−* mice were sacrificed for IF staining as previously described^54^. Excised bladders were fixed in 4% (wt/vol) paraformaldehyde for 2 hours at room temperature. Fixed tissue was cryoprotected, frozen, sectioned, and incubated with rabbit polyclonal anti-P2X_4_ antibody (Catalogue #: APR002, Alomone lab), polyclonal sheep anti-ENTPD2 antibody (Catalogue #: AF5797, R&D systems, Minneapolis, MN), monoclonal PDGFRα affinity purified goat IgG antibody (Catalogue #: AF1062, R&D system, Minneapolis, MN), chicken polyclonal anti-PGP9.5 antibody (Catalogue #: ab72910, ABCAM, Cambridge, MA), and purified rat anti-mouse β1 integrin antibody (Catalogue #: 550531, BD Bioscience, San Jose, CA) (1:100 dilution) overnight at 4 °C. The sections were then incubated with a mixture of Alexa 488-conjugated secondary antibody (diluted 1:100), Alex 546-conjugated secondary antibody (diluted 1:100), and Topro-3 (1:1,000). Imaging was performed on a Zeiss LSM-510 confocal microscope equipped with argon and green and red helium-neon lasers (Thornwood, NY). Images were acquired by sequential scanning with a 63X (1.4 numerical aperture) planapochromat oil objective. The images (512 & 512 pixels) were saved as TIFF files, and were imported into Adobe Illustrator CS3. In the current study, each bladder was sectioned to obtain 2 slides with 4–5 sections of tissue on each slide. Each tissue section was examined under the microscope to ensure the consistency of the staining result, and representative images were taken.

### Spontaneous voiding spot assay (VSA)

VSA’s were performed as described previously^55,56^. Male mice can exhibit dominant and territorial marking behaviour, therefore only female mice were used in this experiment. Individual mice were gently placed in a standard polycarbonate mouse cage with Blicks Cosmos Blotting Paper (Cat #10422-1005) placed in the bottom, for 4 hours. Mice were given standard dry mouse chow for the duration of the assay. Water was withheld due to problems created by water dripping onto the filter paper. After 4 hours mice were returned to their home cages and the filter paper was allowed to dry. Filters were photographed under ultraviolet light at 365 nm in a UVP Chromato-Vue C-75 system (UVP, Upland, CA) that incorporates an onboard Canon digital single lens reflex camera (EOS Rebel T3 – 12 megapixels). Overlapping voiding spots were visually examined and manually separated by outlining and copying, then pasting to a nearby empty space in ImageJ software (http://fiji.sc/wiki/index.php/Fiji). Images were analyzed by UrineQuant software developed by us in collaboration with the Harvard Imaging and Data Core. The results table, which contains the area of each voiding spot and total number of spots, were imported into Excel software for further statistical processing. A volume:area standard curve on this paper determined that 1 mm^2^ is equal to 0.283 µl of urine. Voiding spots that have an area ≥ 80 mm^2^ are considered to be primary voiding spots (PVS)^55^.

### Cystometrograms (CMG)

CMG was performed as described previously and only female mice were used in this experiment.^55,56^. Mice were anesthetized by subcutaneous injection of urethane (1.4 g/kg). Once the pedal reflex was absent, a 1 cm midline abdominal incision was performed and a flame flanged PE50 tubing was implanted through the dome of the bladder, which was secured in place with an 8–0 silk surgical suture. The mouse was placed into a restrainer and the catheter was connected to a pressure transducer (and syringe pump by side arm) coupled to data acquisition devices (WPI Transbridge [Sarasota, FL] and AD Instruments Powerlab 4/35 [Colorado Springs CO]) and computerized recording system (AD Instruments LabChart software).

### Statistical analyses

Data are presented as mean ± standard deviation (SD). Data were analysed using Student’s *t*-test for paired groups or one-way analysis of variance (ANOVA) for comparison among groups. Bonferroni’s multiple comparison post-hoc tests were used where necessary and P < 0.05 was considered to be significant.

## Electronic supplementary material

supplemental information
