# Supplementary material for: Role of P2X4 Receptor in Mouse Voiding Function
Source: Sci Rep. 2018 Jan 30;8:1838. doi: 10.1038/s41598-018-20216-4 (PMC5789870; doi:10.1038/s41598-018-20216-4)
Supplement: Supplementary file 1 — supplemental information [file 41598_2018_20216_MOESM1_ESM.doc]

**Supplemental information**

**Role of P2X_4_ Receptor in Mouse Voiding Function**

Weiqun Yu*, Warren G. Hill, Simon C. Robson, and Mark L. Zeidel

Department of Medicine, Beth Israel Deaconess Medical Center and Harvard Medical School, Boston, Massachuesetts.

Supplemental figure 1

Supplemental figure 1. Anti-P2X4 antibody from Enzo Life Sciences did not distinguish between wild type and *P2X_4_-/-* mice bladder tissue. Mouse bladder lysates were resolved by SDS-PAGE, and Western blots were probed with anti-P2X_4_ antibody (Enzo Life Sciences: catalogue #: Alx-215-033-R100). Results indicate that this antibody detects several non-specific protein bands in both wildtype and *P2X_4_-/-* mice bladder tissue (A); Cryosections of mouse (wild type and *P2X_4_-/-*) bladders were labeled with anti-P2X_4_ antibody (Enzo Life Sciences: catalogue #: Alx-215-033-R100, green), and Topro-3 to label nuclei (blue). Results indicate that this antibody can label BSM in both wildtype (B) and *P2X_4_-/-* (C) mice bladder. Scale bar = 10 µm.

Supplemental figure 2

Supplemental figure 2. Anti-P2X_4_ antibody has non-specific cross reactivity with nerve fibers. Cryosections of *P2X_4_-/-* mouse bladders were labeled with anti-P2X_4_ antibody (Alomone lab: catalogue #: APR002, green), anti-PGP9.5 (A), anti-β1 integrin (B), and anti-PDGFRα (C) antibodies (red), and Topro-3 to label nuclei (blue). Results indicate that non-specific anti-P2X_4_ positive thin fibrous structures in the P2X_4_ knockout co-localize with PGP9.5 but not with β1 integrin and PDGFRα, indicating a cross reactivity of this anti-P2X_4_ antibody with other proteins in nerve fiber, possibly P2X_2/3_. Scale bar = 10 µm.
